# Efficacy and safety of different regimens in the treatment of patients with latent tuberculosis infection: a systematic review and network meta-analysis of randomized controlled trials

**DOI:** 10.1186/s13690-023-01098-z

**Published:** 2023-05-05

**Authors:** Dawit Getachew Assefa, Ahmed Bedru, Eden Dagnachew Zeleke, Solomon Emiru Negash, Dejene Tolossa Debela, Wondowsen Molla, Nebiyu Mengistu, Tigist Tekle Woldesenbet, Neway Fekede Bedane, Violet Dismas Kajogoo, Mary Gorret Atim, Tsegahun Manyazewal

**Affiliations:** 1 KNCV Tuberculosis Foundation, Country Office, Addis Ababa, Ethiopia; 2 Department of Midwifery, Bule Hora University, Bule Hora, Ethiopia; 3 Department of Emeregency, Nashville Veterans Affairs Medical Center, Nashville, USA; 4 Addis Ababa University, College of Health Sciences, Center for Innovative Drug Development and Therapeutic Trials for Africa (CDT-Africa), Addis Ababa, Ethiopia; 5 Department of Midwifery, Dilla University, Dilla, Ethiopia; 6 Department of Psychiatry, Dilla University, Dilla, Ethiopia; 7 Department of Public Health, Hawassa University, Hawassa, Ethiopia; 8 Tanzania Diabetes Association, Dar Es Salaam, Tanzania; 9 Mafia District Hospital, Mafia Islands, Tanzania; 10 Department of Public Health, Faculty of Health Sciences, Busitema University, Mbale, Uganda

**Keywords:** Systematic review, Network meta-analysis, Tuberculosis preventive therapy

## Abstract

**Background:**

Treatment of latent tuberculosis infection (LTBI) is effective in preventing progression to TB disease. This study aimed to synthesize available evidence on the efficacy, adherence, and safety of LTBI treatment in order to assist policymakers to design appropriate national treatment policies and treatment protocols.

**Method:**

The PRISMA-NMA was used to review and report this research. Randomized controlled trials which compared the efficacy and safety of LTBI treatments were included. A systematic literature search was done to identify relevant articles from online databases PubMed/ MEDLINE, Embase, and Cochrane Center for Clinical Trial database (CENTRAL). The network meta-analysis was done using R- studio Version 1.4.1103.

**Result:**

In this review, 42 studies were included, which enrolled 46,022 people who had recent contact with patients with active tuberculosis, evidence radiological of previous tuberculosis, tuberculin test equal or greater than 5 mm, radiographs that indicated inactive fibrotic or calcified parenchymal and/or lymph node lesions, had conversion to positive results on a tuberculin skin test, participants living with HIV, chronic Silicosis, immigrants, prisoners, old people, and pregnant women who were at risk for latent TB were included.

The incidence of TB among people living with HIV who have taken 3RH as TPT was lower, followed by 48%,followed by 6H (41%). However, 3HP has also the potential to reduce the incidence of TB by 36% among HIV negative patients who had TB contact history. Patients’ adherence to TPT was higher among patients who have taken 4R (RR 1.38 95% CI 1.0,1.89) followed by 3RH (34%). The proportion of subjects who permanently discontinued a study drug because of an adverse event were three times higher in the 3RH treatment group. Furthermore, the risk of grade 3 and 4 liver toxicity was significantly higher in 9H followed by 1HP, and 6H.

**Conclusion:**

From this review, it can be concluded 3RH and 6H has a significant impact on the reduction of TB incidence among PLWH and 3HP among HIV negative people who had TB contact history. However, combinations of rifampicin either with isoniazid were significantly associated with adverse events which resulted in permanent discontinuation among adult patients. Furthermore, grade 3 and 4 liver toxicity was more common in patents who have taken 9H, 1HP, and 6H. This may support the current recommended TPT regimen of 3HP, 3RH, and 6H.

## Introduction

Tuberculosis (TB) remains the leading cause of morbidity and mortality from a single infectious disease [1], with one-fourth of the global population, approximately 2 billion persons, estimated to be infected with TB [2, 3]. The occurrence of latent tuberculosis infection (LTBI)—a state of persistent immune response to stimulation by *Mycobacterium tuberculosis* antigens with no evidence of clinically manifest active TB – is impeding the effort to prevent and control TB [2, 4-6]. Treatment of LTBI is effective in preventing progression to TB disease while approximately 5%–10% of persons with LTBI progress to active TB disease if untreated [7-9]. The probability of progression to active TB disease is higher in specific risk groups including people living with HIV, receiving dialysis, preparing for an organ or hematological transplant, prisoners, health workers, immigrants, homeless, silicosis, diabetes, and drug addicted [10-14].

WHO recommends TB preventive treatment (TPT) a key approach to end TB. The current TPT treatment consists of the preferred three rifamycin-based preferred regimens: 3 months of once-weekly isoniazid plus rifapentine, 4 months of daily rifampin, or 3 months of daily isoniazid plus rifampin [3, 4]. Some studies reported that rifamycin-based regimens are effective and safe for treatment of LTBI, with higher treatment completion rates [15-20]. Some other studies reported that regimens isoniazid monotherapy, daily for 6–9 months, is efficacious but with a higher risk of toxicity and a lower treatment completion rates compared to rifamycin-based regimens with shorter treatment durations [21, 22]. Studies also demonstrated that 3 months of once-weekly isoniazid plus rifapentine is non-inferior to other regimens, but with slightly higher adverse events [21-25]. A recent study also showed a beneficial effect of one month daily isoniazid and rifapentine combination-therapy [26]. There are concerns about pragmatic and long-term aspects of TPT, including adherence, potential emergence of drug resistance, and cost-effectiveness in resource-constrained settings [27]. Several systematic reviews as well as network meta-analyses have yet been documented about treatment of LTBI [8, 28-30]; however, majority focused on effectiveness of different regimens that did not provide information about the indirect relative (comparision of different TPTs with one another indirectly) safety of the TPT regimens. Also, these studies have provided separate information about the efficacy of TPT among people living with HIV, immigrants, children, and people who had Tb cotact history. We belive that there is a need for an updated and more comprencive evidence regarding the efficacy and safety of TPTs for different population groups.

We, therefore, conducted this systematic review and network meta-analysis of randomized controlled trials (RCTs) using the frequentist model to provide an up-to-date summary and analysis of previously published studies that have evaluated LTBI regimens and made informative comparisons of their relative efficacy and adverse event profiles.

## Methods

The protocol for this systematic review and network meta-analysis has been registered at the International Prospective Register of Systematic Reviews (PROSPERO) database, ID: CRD42022334163 [31]. The PRISMA statement extension for systematic reviews incorporating network meta-analysis (PRISMA-NMA) was used to review and report this research [32].

### Eligibility Criteria

- ○ The PICOS format [33] was used to identify eligible studies.

### Participants

- ○ People who had recent contact with patients with active tuberculosis, evidence radiological of previous tuberculosis, tuberculin test equal or greater than 5 mm, radiographs that indicated inactive fibrotic or calcified parenchymal and/or lymph node lesions, had conversion to positive results on a tuberculin skin test, participants living with HIV, chronic Silicosis, immigrants, prisoners, old people, and pregnant women who were at risk for latent TB were included.
- ○ Tuberculosis (TB) contacts are people who have close contact with patients with infectious TB.

### Interventions

- 12 months 600 mg rifamycin plus 300 mg isoniazid (12RH).
- 3 months 600 mg rifamycin plus 300 mg isoniazid (3RH).
- 3 months of once-weekly 900 mg isoniazid plus 900 mg rifapentine (3HP).
- 18 months daily 300 mg isoniazid (18H).
- 72 months daily 300 mg isoniazid (72H).
- 4 months daily 600 mg rifamycin plus 300 mg isoniazid (4RH).
- 6 months daily 300 mg isoniazid plus 800 mg ethambutol (6EH).
- 1 month daily 300 mg isoniazid plus 600 mg rifapentine (1HP).
- 2 months daily 600 mg rifamycin (2R).
- 4 months of once-weekly 900 mg isoniazid plus 600 mg rifapentine (4HP).
- 2 months of twice-weekly 600 mg isoniazid plus 600 mg rifapentine (2HP TW).
- 3 months of once-weekly 900 mg isoniazid plus 900 mg rifapentine (3HP).
- 4 months daily 600 mg rifamycin (4R).
- 3 months daily 600 mg rifamycin (3R).
- 6 months daily 300 mg isoniazid (6H)
- 9 months daily 300 mg isoniazid (9H)
- 12 months daily 300 mg isoniazid (12H)
- 18 months daily 300 mg isoniazid (18H)
- 24 months daily 300 mg isoniazid (24H)
- 72 months daily 300 mg isoniazid (72H)

### Comparator

- 3 months of once-weekly 900 mg isoniazid plus 900 mg rifapentine (3HP), or
- Placebo

### Outcome measures

#### Primary outcomes

- Treatment efficacy, thus the overall incidence of TB among all kinds of participants, PLWHIV, and HIV negative participants who have taken TPT.

#### Secondary outcomes

- ○ Adverse event including serious adverse event was assessed.
- ○ Adherence to medications
- ○ The incidence of TB in patients living with chronic silicosis.

### Studies

RCTs published from 1993–2022, involving participants of any age group which compared the efficacy, safety, or adherence of LTBI regimens and exploratory analysis of data from RCTs. Those studies conducted and published before 1993 were either not freely available for access or not aligned with the current WHO INH recommended dose (300 mg/day). Regimens containing PZA were not considered among those of primary interest due to their poor toxicity profile. Two reviwers assessed the titles and abstracts from the primary search independently. Those seemingly meeting inclusion criteria were further assessed by review of full texts by the same two reviewers. Disagreements were resolved by consensus.

### Electronic searches

A systematic literature search was done to identify relevant articles from online databases PubMed/ MEDLINE, Embase, and Cochrane Center for Clinical Trial database (CENTRAL). To search and assess ongoing or unpublished trials, ClinicalTrials.gov and the WHO International Clinical Trials Registry Platform, and the US Food and Drug Administration (FDA) were searched. The search was done according to guidance provided in the Cochrane Handbook for Systematic Reviews of Interventions [33].

The search strategies in PubMed for the MeSH terms and text words were “Tuberculosis” [MeSH Terms]) OR “Latent Tuberculosis Prevention” [MeSH]) AND “Tuberculosis Prevention therapy” [MeSH]) AND “isoniazid” [MeSH]) AND “rifapentine” [MeSH]) AND “rifamycin” [MeSH]) AND “ethambutol” [MeSH]).

### Study selection, data collection, and data analysis

We used the Cochrane Handbook for Systematic Reviews of Interventions [34], the R- studio Version 1.4.1103, and the EndNote X7 for data management and analysis. Two authors independently reviewed the results and disagreements resolved through discussion. When clarification was necessary, the trial authors were contacted.

### Data extraction and management

The title and abstract were produced from the electronic search and independently screened by two authors based on RCTs that were LTBI. The information collected were trial characteristics including methods, participants, interventions, and outcomes as well as data on dose and drug ratios of the combinations. Relevant information such as title, name of the journal, year of publication, author’s first name, country, type of participant, age, sex, randomization, post treatment follow up time, methody of study drug adminstartion (directly observed therapy (DOT) or self administration), publication status, study design, study setting, follow-up period, sample size, funding source, baseline characteristics of study subjects, adherence, TB incidence, adverse events, and serious adverse events were extracted from each article using a structured data extraction format adapted from Cochrane. The number of participants randomized and the number analyzed in each treatment group for each outcome were also captured. Two authors independently extracted the data and cross-checked. For dichotomous outcomes, the number of participants experiencing the event and the number of participants in each treatment group were documented.

### Assessment of risk of bias in included studies

The risk of bias for each trial was evaluated by two review authors independently using the Cochrane Collaboration's tool for assessing the ‘Risk of bias’ [33].

### Meta-analysis and network meta-analysis

The network meta-analysis was done using R- studio Version 1.4.1103. The network meta-analysis were performed using the frequentist model for each treatment comparison, using the Netmeta package. To indentify which treatment has the highest effects, the netrank function implemented in {netmeta} used. It allowed us to generate a ranking of treatments, indicated which treatment was more or less likely to produce the largest benefits. This frequentist method uses P-scores to rank treatments, which measure the certainty that one treatment is better than another treatment, averaged over all competing treatments.

### Geometry of network

Network geometry used nodes to represent different LTBI treatments and edges to represent the head-to-head comparisons between network nodes. The nodes' size and edge thickness were represented sample sizes of intervention and numbers of included trials, respectively. The network nodes were categorized as follows: 1. 6H, 2. 9H, 3. 12H, 4. 24H, 5. 3HP, 6. 3RHZ, 7. 4HP TW, 8. 2RZ, 9. 2R, 10. 3R, 11. 4R 12. 2HP TW, 13. 1HP, 14. 6EH, 15. 4RH, 16. 72 H, 17. 18H, 18. 3RH, 19, 12RH, and 20. Placebo.

### Description of network diagram

**Fig. 1:**
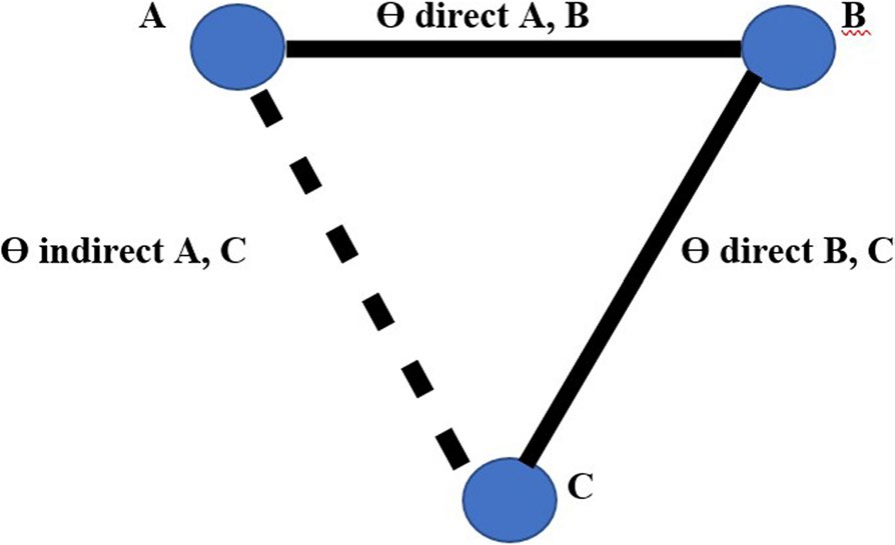
Network diagram

Imagine that we have extracted data from some randomized controlled trial i, which compared the effect of treatment A to another condition B. Our graph has two core components. The first one are two circles (so-called nodes), which represent the two conditions A and B in trial i. The second component is the line connecting these two nodes. This line is called an edge. The edge represents how A and B relate to each other. Also, imagine that we have also obtained data from another study j. This trial also used the control condition B. But instead of administering A, this study used another treatment C. In study j, treatment C was also compared to B (Fig. 1).

It is clearly visible that the graph now contains two effect size estimates: θ^^i,A,B^, comparing A to B, and θ^^j,C,B^, the comparison between C and B. Since both of these effect sizes were directly observed in “real” trials, we call such information direct evidence. Therefore, we denote these effect sizes with θ^^directB,A^ and θ^^direct B,C^. The B condition (our control group) is directly connected to all other nodes. It takes only one “step” in the graph to get from B to the two other nodes A and C: B → A, B → C. In contrast, A and C only have one direct connection, and they both connect to B: A → B and C → B (Fig. 1).

However, there is an indirect connection between A and C. This connection exists because B serves as the link, or bridge, between the two conditions: A → B → C. As a result, there is indirect evidence for the relationship between A and C, which can be derived from the structure of the network (Fig. 1).

Using information from the directly observed edges, we can calculate the effect of the indirectly observed comparison between A and C. We denote this non-observed, indirect effect size with θ ^^ indirect A,C^. Furthermore, we can see that the edges in the plot have a different thickness. The degree of thickness represents how often we find a specific comparison in our network Fig. 1.

### Assessment of heterogeneity

Heterogeneity among the included trials was assessed by inspecting the forest plots and the Cochrane Q and I^2^ statistic was used to measure heterogeneity among the trials in each analysis, the Chi^2^ test with a *P* < 0.10 to indicate statistical significance was used.

To further determine evaluate inconsistency in our network model, Net heat plots was done. The gray boxes signify how important a treatment comparison is for the estimation of another treatment comparison. The bigger the box, the more important the comparison. The colored backgrounds signify the amount of **i**nconsistency of the design in a row that can be attributed to the design in a column**.** Field colors can range from a deep red (which indicates strong inconsistency) to blue (which indicates that evidence from this design supports evidence in the row).

Another method to check for consistency in our network is net splitting. This method splits our network estimates into the contribution of direct and indirect evidence, which allows us to control for inconsistency in the estimates of individual comparisons in our network. When a difference is *p* < 0.05, there is a significant disagreement (inconsistency) between the direct and indirect estimate.

### Result

**Fig. 2:**
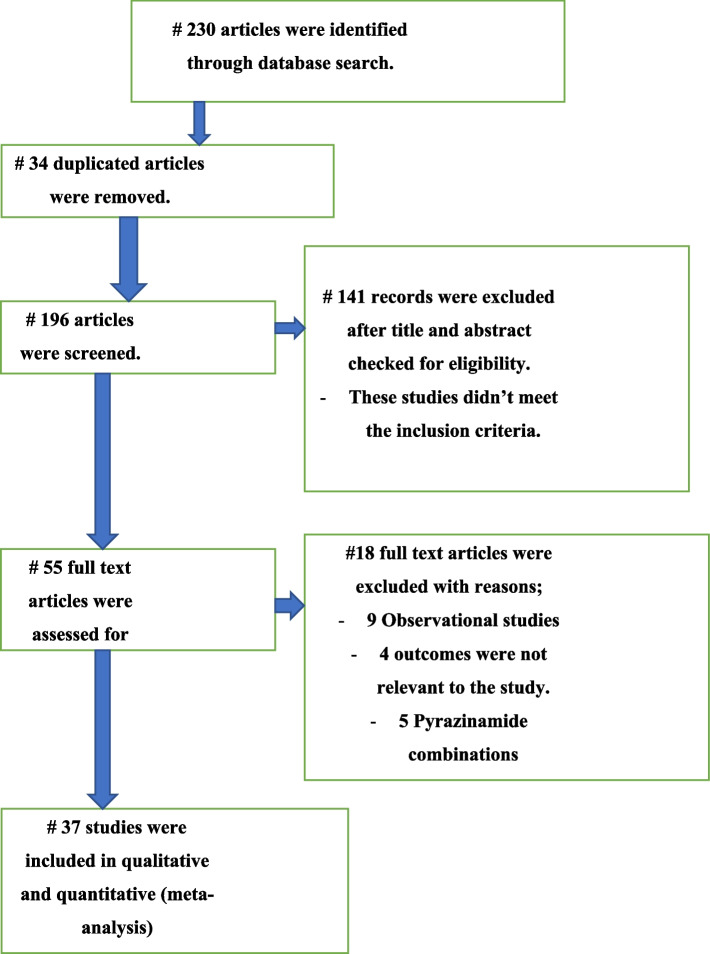
PRISMA study flow diagram of the RCTs published between 1993–2022

The search resulted in a total of 320 studies, of which 55 full-text eligible studies were evaluated further and 37 of them fulfilled the inclusion criteria and included in the network meta-analysis and qualitative analysis (Fig. 2).

### Characteristics of included studies

In this review, 37 studies were included, which enrolled 46,022 participants living with HIV, chronic Silicosis, had contact history with TB infected person, immigrants, prisoners, old people, and pregnant women who were at risk for latent TB were included in Table 1.

**Table 1:** Characteristics of included RCTs published between 1993–2022

Study ID	Country	Type of patients	Age				Sex		Randomization								PTFT	Rx Mode
							**Male**	**Female**										
Pape, 1993 [35]	Haiti	PLWHIV	12H		Placebo		27	91	12H	Placebo							2 years	N/A
			Mean 31.1 years		Mean 30.6 years				58	60								
Cowie, 1996 [36]	Canada	Silicosis	RHZ		Placebo		382	0	3RHZ	Placebo							4 years	DOT
			Mean 47.0 years		Mean 47.3 years				191	191								
Gordin, 1997 [37]	USA	PLWHIV	6H		Placebo		322	195	6H	Placebo							2 ½ years	N/A
			Mean 37.4 years		Mean 38.2 years				260	257								
Hawken, 1997 [11]	Kenya	PLWHIV	6H		Placebo		269	415	6H	Placebo							2 years	Self administered
			Mean 31.1 years		Mean 31.1 years				342	342								
Whalen, 1997 [38]	Uganda	PLWHIV	6H	3RH	3RHZ	Placebo	854	1882	6H	3RH	3RHZ					Placebo	2 ½ years	Self administered
			Mean 29 years	Mean 29 years	Mean 29 years	Mean 30 years			931	556	462					787		
			Mean 31 years		Mean 31 years				370	380								
Alfaro, 2000 [39]	Spain	PLWHIV	12H		3RH		102	31	12H	3RH							2 years	Self administered
			Mean 32.2 years		Mean 32.2 years				64	69								
Fitzgerald, 2000 [40]	Haiti	PLWHIV	6H		Placebo		111	116	6H	Placebo							2 years	Self administered
			Mean 32 years		Mean 31.5 years				119	114								
HKCS, 2001 [41]	China	Silicosis	6H	3RH	3R	Placebo	N/A	N/A	6H	3RH		3R			Placebo		2–5 years	Self administered
			N/A	N/A	N/A	N/A			173	167		172			167			
Johnson, 2001 [15]	Uganda	PLWHIV	6H	3RH	3RHZ	Placebo	N/A	N/A	6H	3RH		3RHZ			Placebo		5 years	Self administered
			N/A	N/A	N/A	N/A			931	556		462			787			
Quigley, 2001 [42]	Zambia	PLWHIV	9H	3RZ	Placebo		47	53	9H	3RZ		Placebo					3–7 years	Self administered
			N/A	N/A	N/A				26	46		28						
			N/A		N/A													
Menzies, 2004 [43]	Canada	Households contacts	4R		9H		55	51	4R	9H							2 years	Self administered
			Mean 32.9 years		Mean 34.8 years				58	58								
			Mean 37.7 years		Mean 37.0 years				206	193								
Zar, 2006 [44]	South Africa	PLWHIV	6H		Placebo		145	118	6H	Placebo							2 years	N/A
			Median 29.6 months		Median 22.1 months				132	131								
Geijo, 2007 [45]	Spain	Households contacts and evidence radiological of previous tuberculosis	6H		3RH		53	43	6H	3RH							5 years	N/A
			Median 44.16 years		Median 41.38 years				45	51								
Mohammed, 2007 [46]	South Africa	PLWHIV	12H		Placebo		58	60	12H	Placebo							2 years	Patient nominated suppervisor
			Mean 39.7 years		Mean 37.8 years				68	50								
Rivero, 2007 [47]	Spain	PLWHIV	6H	3RH	2RZ		251	61	6H	3RH	2RZ						2 years	self administered
			Median 31.3 years	Median 33.0 years	Median 33.0 years				108	103	105							
Spyridis, 2007 [48]	Greek	Households contacts and evidence radiological of previous tuberculosis	4RH	3RH	9H		476	450	4RH	3RH	9H						≧ 3 years	self administered
			Mean 9.1 years	Mean 8.8 years	Mean 7.9 years				474	220	232							
Menzies, 2008 [20]	Canada, Saudi Arabia, and Brazil	Households contacts	4R		9H		446	400	4R	9H							1 year	N/A
			N/A		N/A				420	427								
Trajman, 2009 [49]	Canada, Brazil, and Saudi Arabia	Households contacts	Median 33 years				N/A	N/A	4R	9H							4 months	self administered
									420	427								
Madhi, 2011 [50]	South Africa	PLWHIV and HIV exposed children	24H		Placebo		648	705	24H	Placebo							2 years	N/A
			Median 96.5 weeks		Median 95.5 weeks				676	677								
Martinson, 2011[51]	South Africa	PLWHIV	3HP	3RH	72H	6H	192	956	3HP	3RH	72H			6H			≦ 6 years	DOT & self administered
			Median 30.3 years	Median 30.5 years	Median 30.2 years	Median 30.4 years			329	329	164			328				
Sterling, 2011 [21]	USA, Canada, Brazil, and Spain	Households contacts and evidence radiological of previous tuberculosis	3HP		9H		4214	3517	3HP	9H							33 months	self administered
			Mean 36		Mean 35				3986	3745								
Chan, 2012 [52]	Taiwan	Prison inmates	6H		4R		373	0	6H	4R							2 months	DOT
			N/A		N/A				183	190								
Fuentes, 2012 [19]	Spain	Immigrant population	6H		3RH		400	190	6H	3RH							5 years	self administered
			Mean 26.1 years		Mean 26.1 years				294	296								
Swaminathan, 2012 [53]	India	PLWHIV	6EH		36H		263	420	6EH	36H							3.5 years	self administered
			Mean 29.9 years		Mean 30.2 years				344	339								
White, 2012 [54]	USA	Prison inmates	4R		9H		339	25	4R	9H							N/A	DOT
			N/A		N/A				180	184								
Gray, 2013 [55]	South Africa	PLWHIV	6H		Placebo		83	84	6H	Placebo							6.6 years	self administered
									85	82								
			Median 32 months		Median 38 months													
Villarino, 2015 [22]	USA, Canada, Brazil, Hong Kong (China), and Spain	Children with recent household contact	3HP		9H		538	520	3HP	9H							33 months	DOT
			Median 10 years		Median 12 Years				552	506								
Sterling, 2014 [24]	USA, Brazil, Spain, Peru, Canada, and Hong Kong	Households contacts and evidence radiological of previous tuberculosis	3HP		9H		N/A	N/A	3HP	9H							1 month	DOT
			N/A		N/A				3893	3659								
Moro, 2016 [23]	USA and Brazil	Households contacts and evidence radiological of previous tuberculosis	N/A		N/A		3352	2563	3HP	9H							3 months	DOT
									3230	3002								
Sterling, 2016 [25]	United States, Brazil, Spain, Peru, Canada, and Hong Kong	PLWHIV	3HP		9H		277	122	3HP	9H							6 years	DOT and self administered
			Median 36 years		Median 36 years				206	193								
Gao, 2018 [16]	China	Older people	Range 50–70 years				2054	1684	3HP	2HP TW			Placebo				2 years	DOT
									1284	1299			1155					
Moro, 2018[56]	USA, Canada, Spain, Peru, and Brazil	Pregnant women	9H		3HP			87	9H	3HP							33 months	DOT and self administered
			Median 25 years		Median 23 Years				56	31								
Sun, 2018 [57]	Taiwan	Households contacts	3HP		9H		152	111	3HP	9H							2 years	DOT
			Mean 31.7 years		Mean 32 years				132	131								
Swindells, 2019 [26]	Africa, Asia, South America, North America, and the Caribbean	PLWHIV	1HP		9H		1386	1614	1HP	9H								
			Median 35 years		Median 35 years				1496	1504							4.5 years	Self administered
Ruan, 2020 [12]	China	Silicosis	Placebo		3HP		N/A	N/A	Placebo	3HP							37 months	DOT
			Median 57 years		Median 57 years				259	254								
Surey, 2021 [58]	United Kingdom	Households contacts	3HP		3RH		26	26	3HP	3RH							2 months	Self administered
			Mean 38.2 years		Mean 32.5 years				27	25								

### Methodological quality and risk of bias

**Fig. 3:**
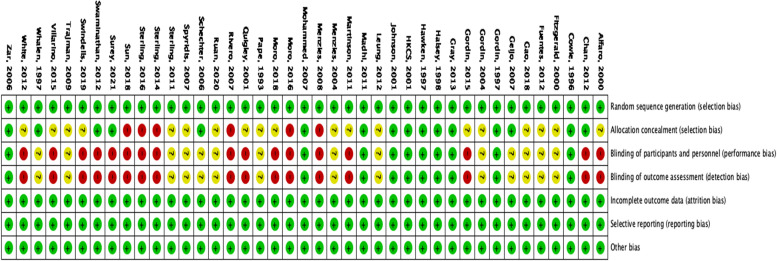
Risk of bias summary: review authors’ judgments about each risk of bias item for each included RCTs published between 1993–2022

Our summary shows that majority of the studies were either open label (high risk for bias) or unclear risk for bias. The rest of the domains were low risk for bias. The’Risk of bias assessments are summarized in Fig. 3**.**

### Overall TB incidence

**Fig. 4:**
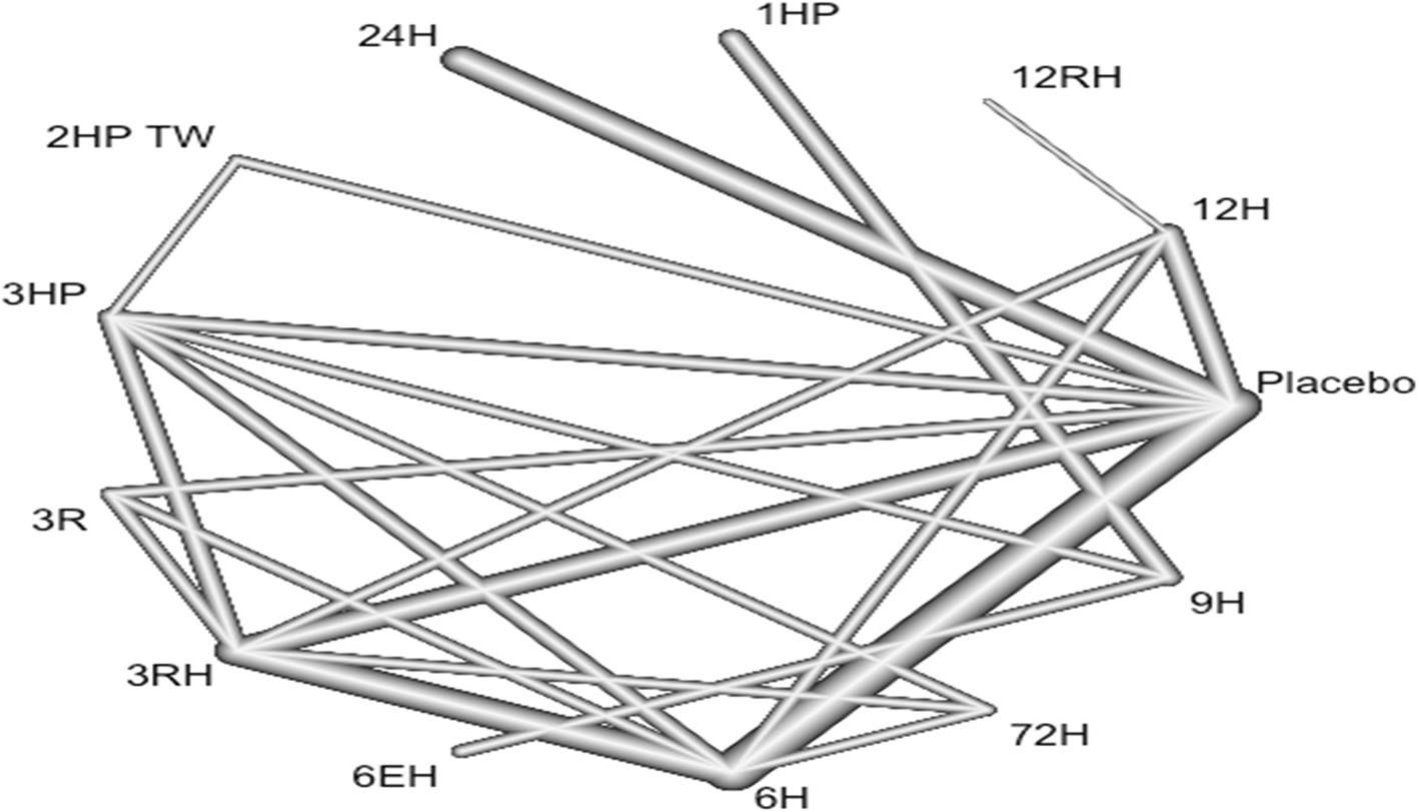
Network diagram for TB incidence among patients who have been taken TPT (RCTs published between 1993–2022)

In this analysis, 29 studies and 15 treatments were included. The test random effect model for heterogeneity (within designs) and inconsistency (between designs) were not statically significant (0.154 and 0.482). The Q value for a full design-by-treatment interaction random effects model also shows that there is no inconsistency (Between designs; Q = 8.79, *P*-value = 0.8445, tau = 0.2714, tau^2^ = 0.0736). The network diagram shows that majority (nine) studies compared placebo with 6 months isoniazid Fig. 4.

**Fig. 5:**
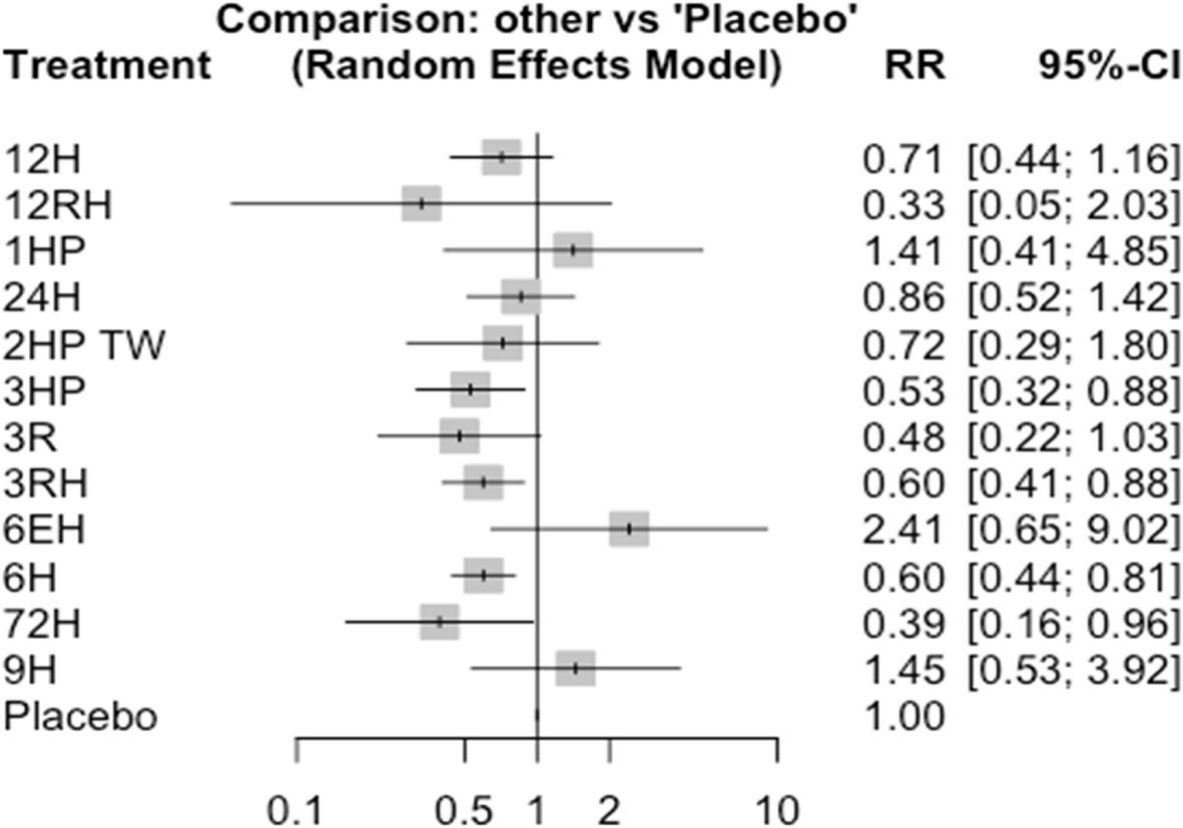
Forest plot TB incidence among patients who have been taken TPT (RCTs published between 1993–2022)

Consistently, the forest plot showed that there are other high-performing treatment regimens beyond the 6 years of continuous isoniazid therapy. The result also showed that some of the confidence intervals are overlapping which makes a clear-cut decision less easy. While 72H, 3HP, 3RH, and 6H significantly reduce the risk of active TB infection by 61%,47%, and 40% respectively compared to placebo (Fig. 5).

### Publication Bias

The funnel plot for incidence of TB was quite symmetrical and this was corroborated by Egger’s test, which was not significant (*p* = *0.2680*).

### Consistency

To further investigate consistency, Net-split has been done and the result shows that there is no significant disagreement between the direct and indirect evidence. Furthermore, the result form the Net-heat plot also shows that the overall consistency of our model is low.

### Incidence of TB among patients living with HIV

**Fig. 6:**
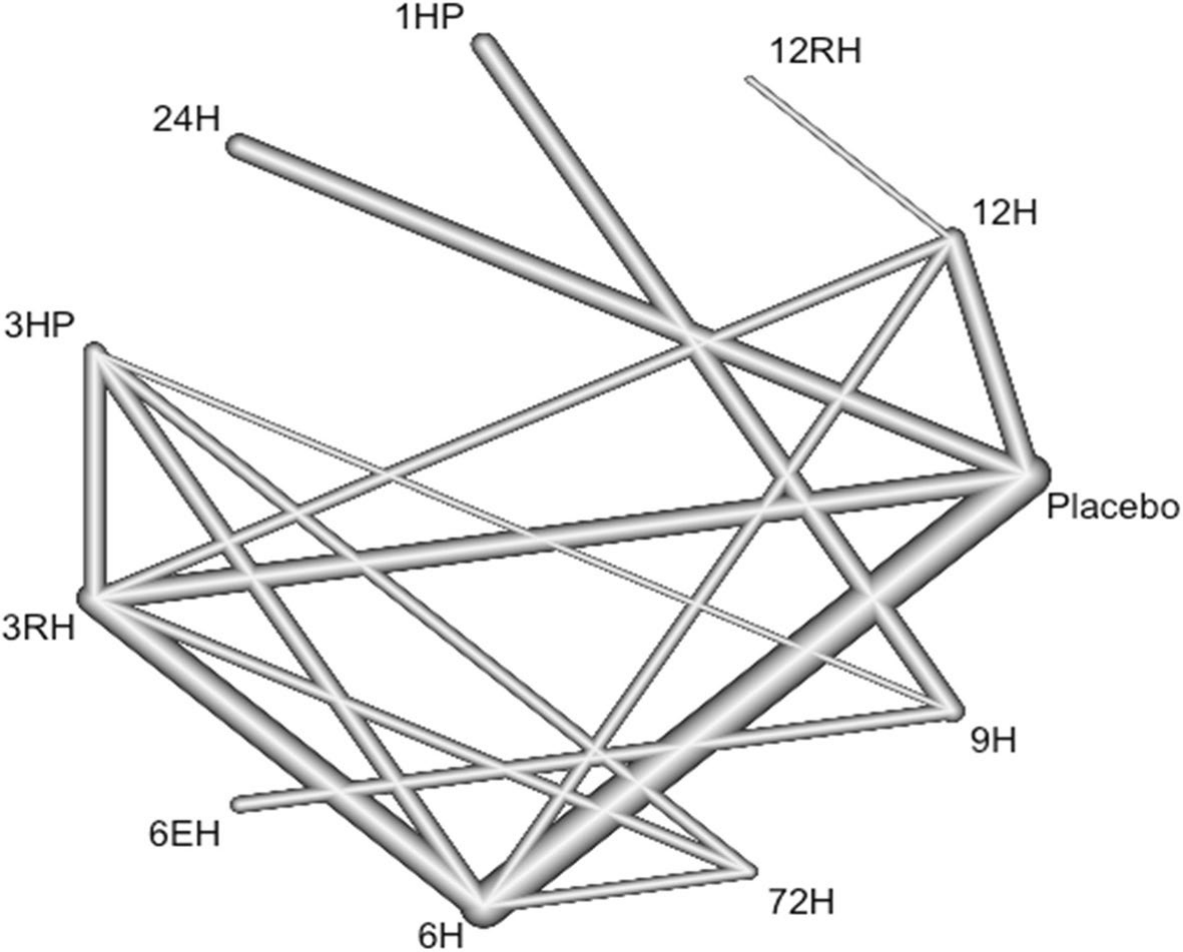
Network diagram for TB incidence among patients living with HIV who have taken TPT (RCTs published between 1993–2022)

In this analysis 17 studies and 11 treatments were included. The heterogeneity/inconsistency in our network model is very low, with t tau^2 = 0.1592; tau = 0.3990; I^2 = 51.8%. Furthermore, the test for inconsistency (between design) was also low (Q = 1.58, *P* = 0.9541). The network diagram shows that majority (seven) studies compared placebo with 6 months isoniazid Fig. 6.

**Fig. 7:**
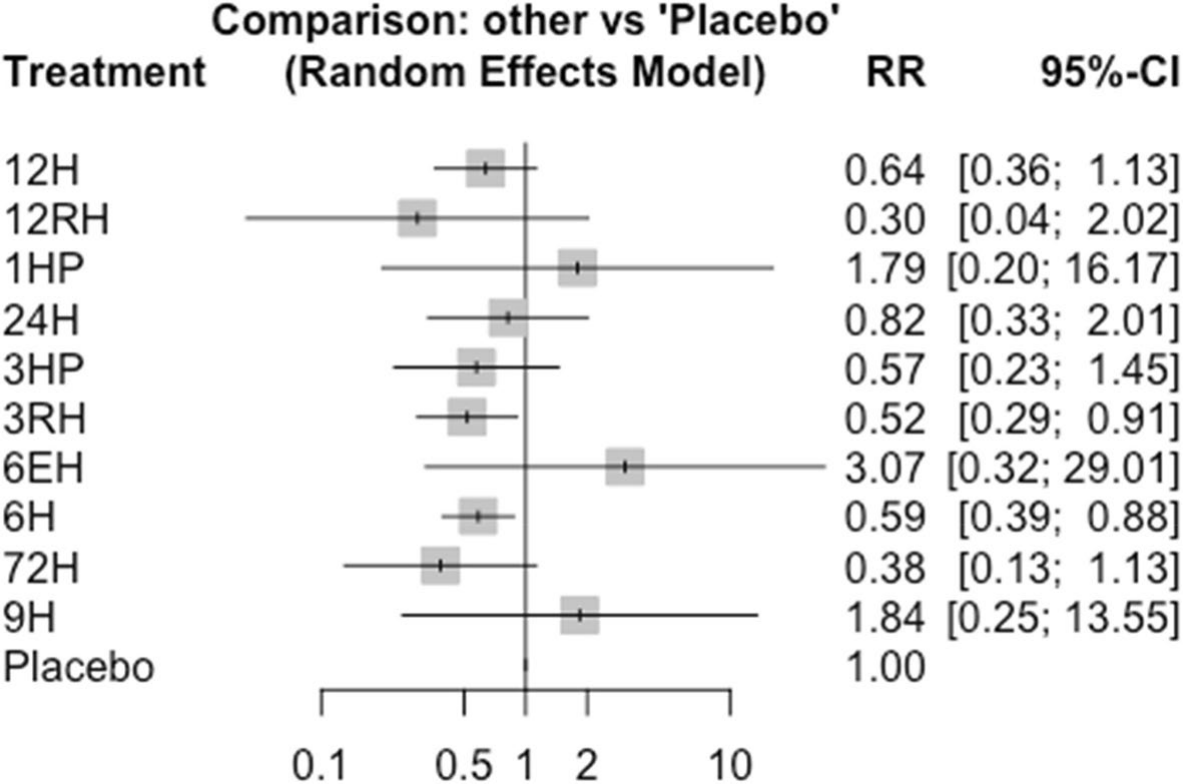
Forest plot for TB incidence among patients living with HIV who have taken TPT (RCTs published between 1993–2022)

The forest plot shows that 3RH, and 6H have shown a significant effect on reducing the incidence of TB among patients living with HIV who have received TPT (RR 0.52 95% CI 0.29–0.91 and RR 0.59 95% CI 0.39–88, respectively) Fig. 7. However, the Net rank shows that 6 months Ethambutol plus Isoniazid have a lowest P-score, which seems this combination therapy is not the best option.

### Consistency

To further investigate consistency, Net-split has been done and the result shows that there is no significant disagreement between the direct and indirect evidence. Furthermore, the result form the Net-heat plot also shows that the overall consistency of our model is low.

### Incidence of TB among HIV-negative patients who had TB contact history

**Fig. 8:**
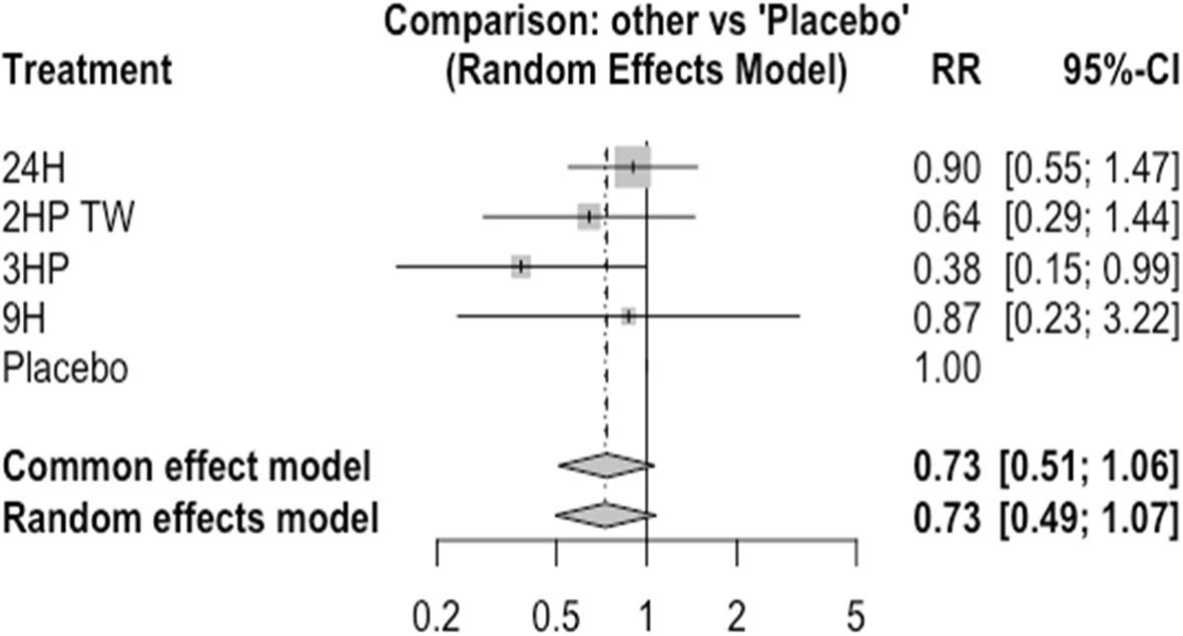
Forest plot for HIV negative patients who had TB contact history (RCTs published between 1993–2022)

**Fig. 9:**
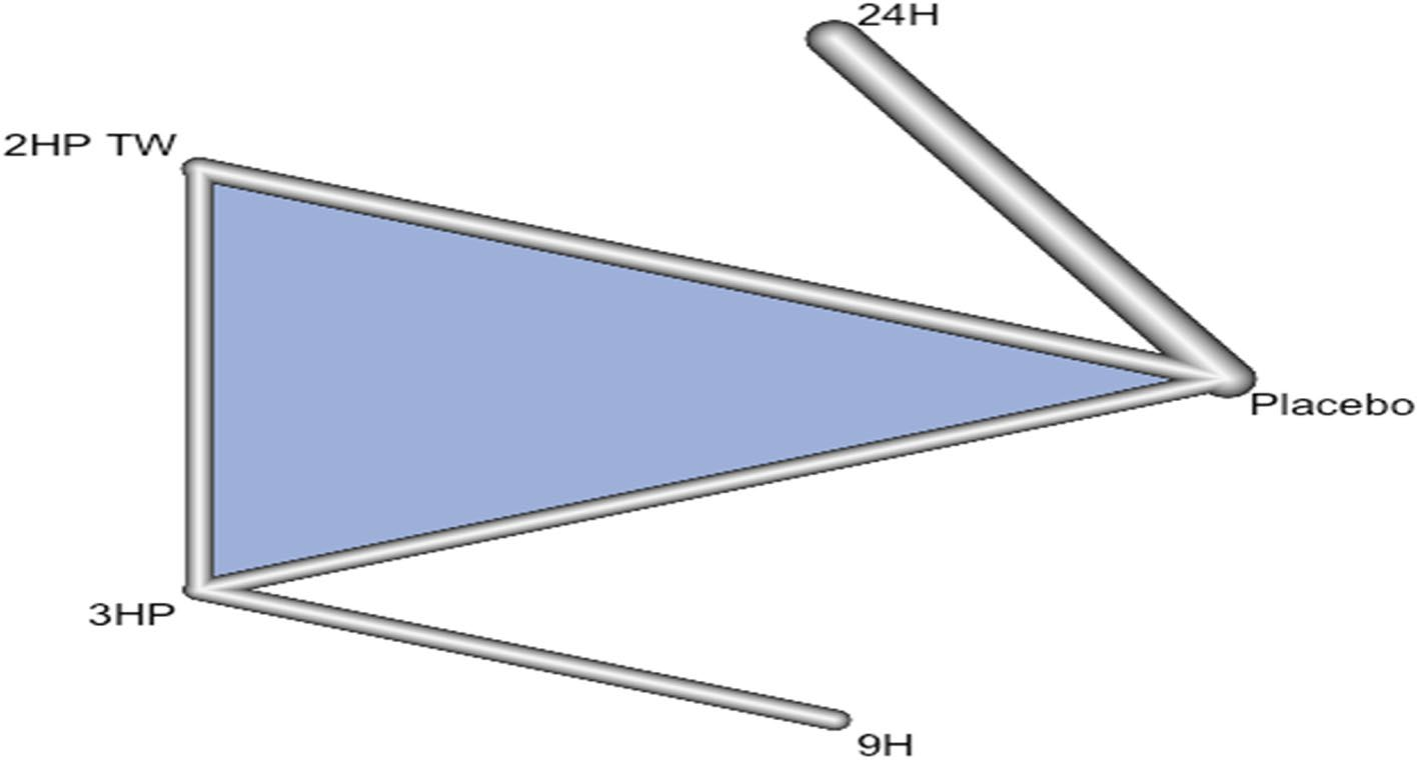
Network diagram for HIV negative patients who had TB contact history (RCTs published between 1993–2022)

In this analysis 3 studies and 5 treatments were included. The forest plot shows that 3HP has a significant benefit over other TB prevention therapies in reducing the incidence of TB among HIV negative patients who had TB contact history Fig. 8. The network diagram shows that more studies compared 24 H and placebo Fig. 9.

### Adherence of patients to TPT

**Fig. 10:**
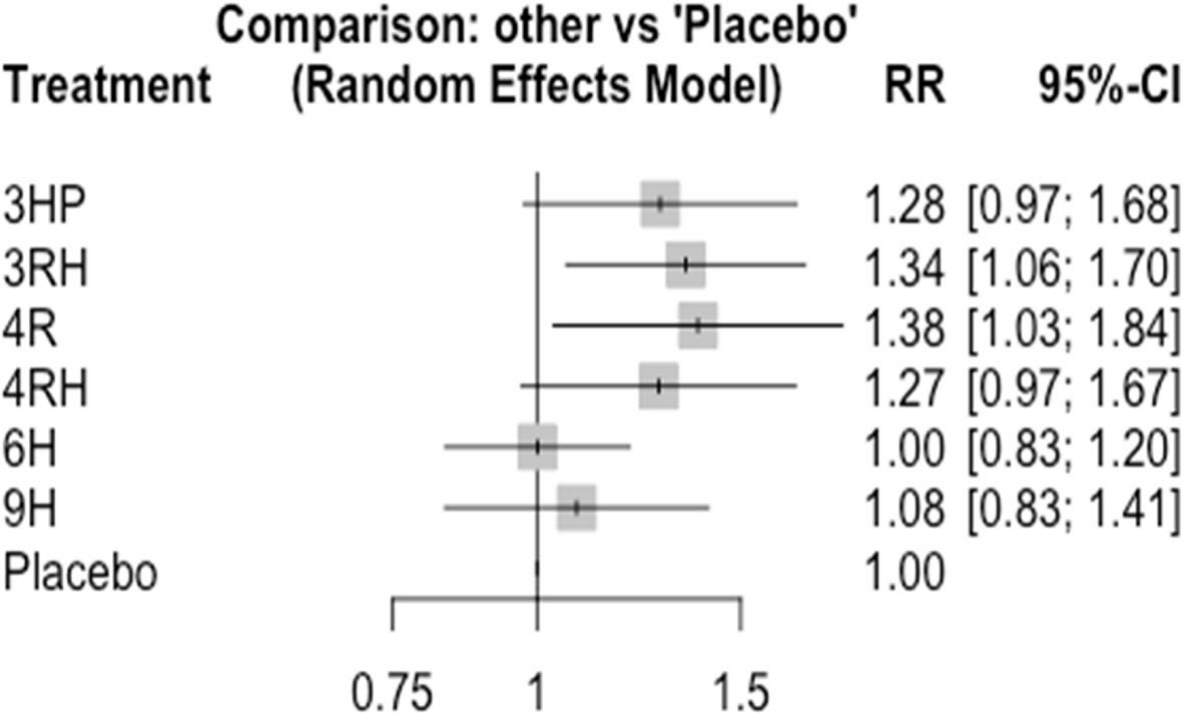
Forest plot of patient’s adherence to TPT* (*RCTs published between 1993–2022)

**Fig. 11:**
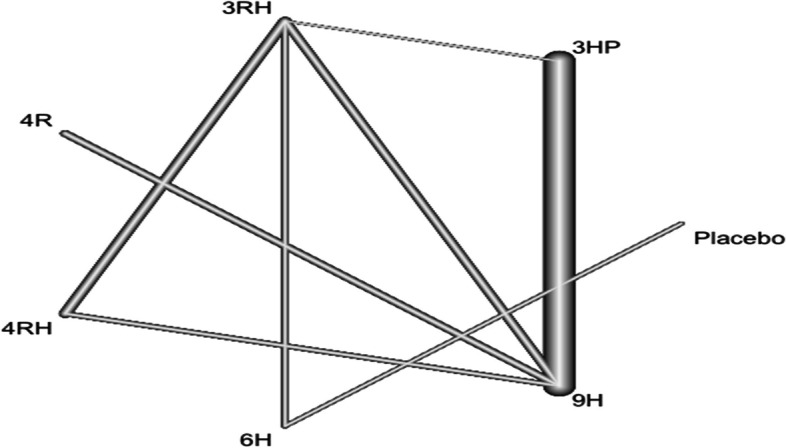
Network diagram for adherence of patients to TPT (RCTs published between 1993–2022)

In this study 14 studies and 6 treatments were involved. The result shows within study heterogeneity was not statistically significant (tau^2^ = 0.0042; tau = 0.0652; I^2^ = 66%). The adherence of TPT was good among patients who have been treated with 4R followed by 3RH Fig. 10. However, patients who were treated with 6H had the least adherence rate compared to patients who were treated with other treatments Fig. 10. The network diagram show that more studies compared 3HP with 9H Fig. 11.

### Adverse events

The included studies [11, 23, 24] have reported peripheral neuropathy as an adverse event and it was more common among patients who have been treated with 6H, 9H, and 3RH. Also, few patients who have been treated with 3R and 4R were also experienced peripheral neuropathy [54]. Furthermore, neutropenia and anemia were also common among patients who have been treated with 9H and 1HP [26]. The proportion of patients who have been experienced headache was higher in 3HP and 3RH [12, 18]. One multi-center study [56] conducted on pregnant women was reported that 12 women experienced fetal loss less that 20 weeks (4 from 3HP and 8 from 9H arms) and two congenital anomalies from 9H arm.

### AE led to treatment discontinuation

**Fig. 12:**
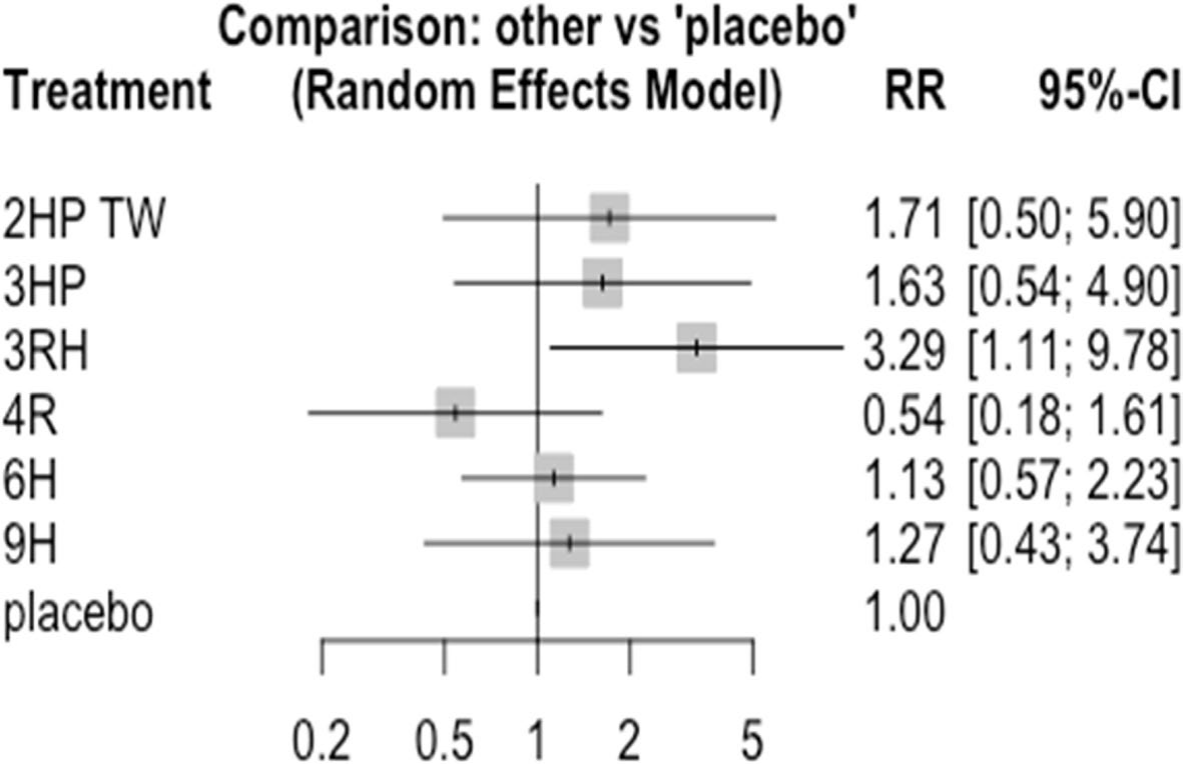
Forest plot of AEs led to treatment discontinuation after TPT initiation (RCTs published between 1993–2022)

**Fig. 13:**
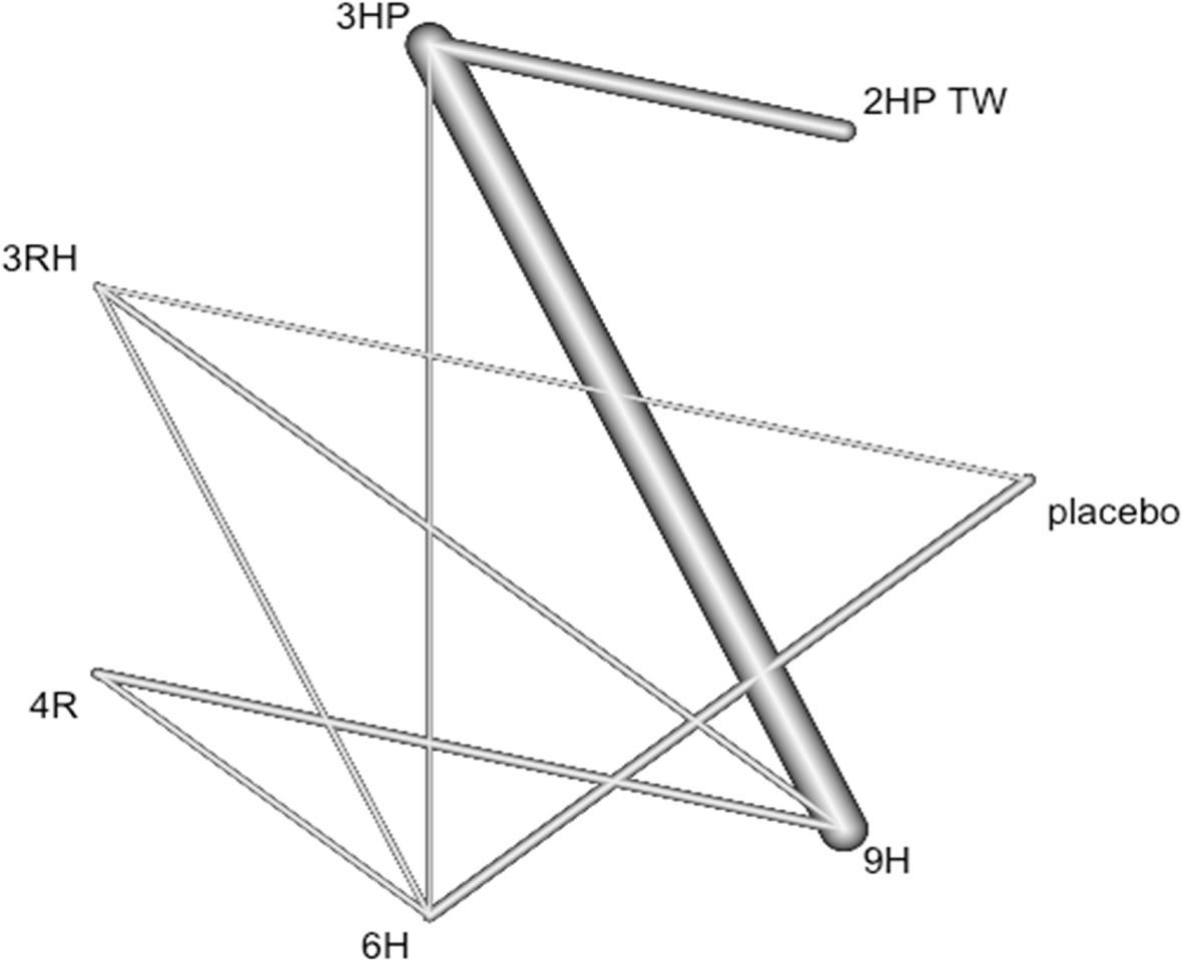
Network diagram of AEs led to treatment discontinuation after TPT initiation (RCTs published between 1993–2022)

In these 11 studies and 7 treatments were included. The forest plot shows that the proportion of subjects who permanently discontinued a study drug because of adverse event were higher in three months daily combination of rifampin and isoniazid (3RH) Fig. 12. However, it was low in patients who have been treated with four months daily rifampin. The network graph shows that most studies compared 3HP with 9H Fig. 13.

### Nausea and vomiting

**Fig. 14:**
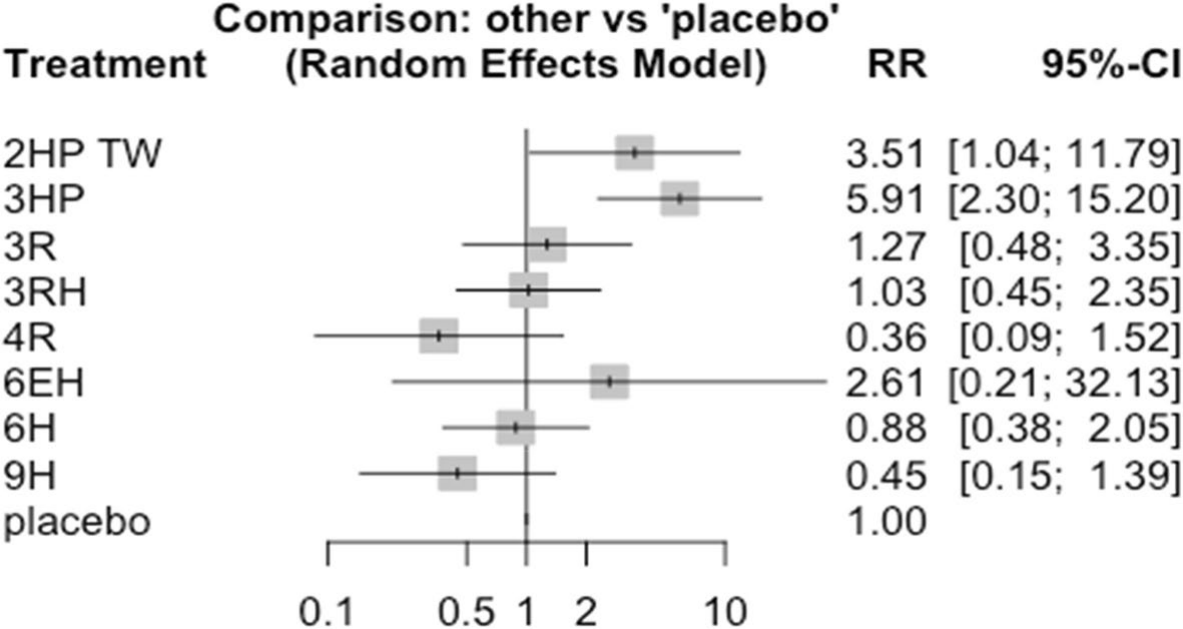
Forest plot for the risk of nausea and vomiting among patients who have taken TPT (RCTs published between 1993–2022)

**Fig. 15:**
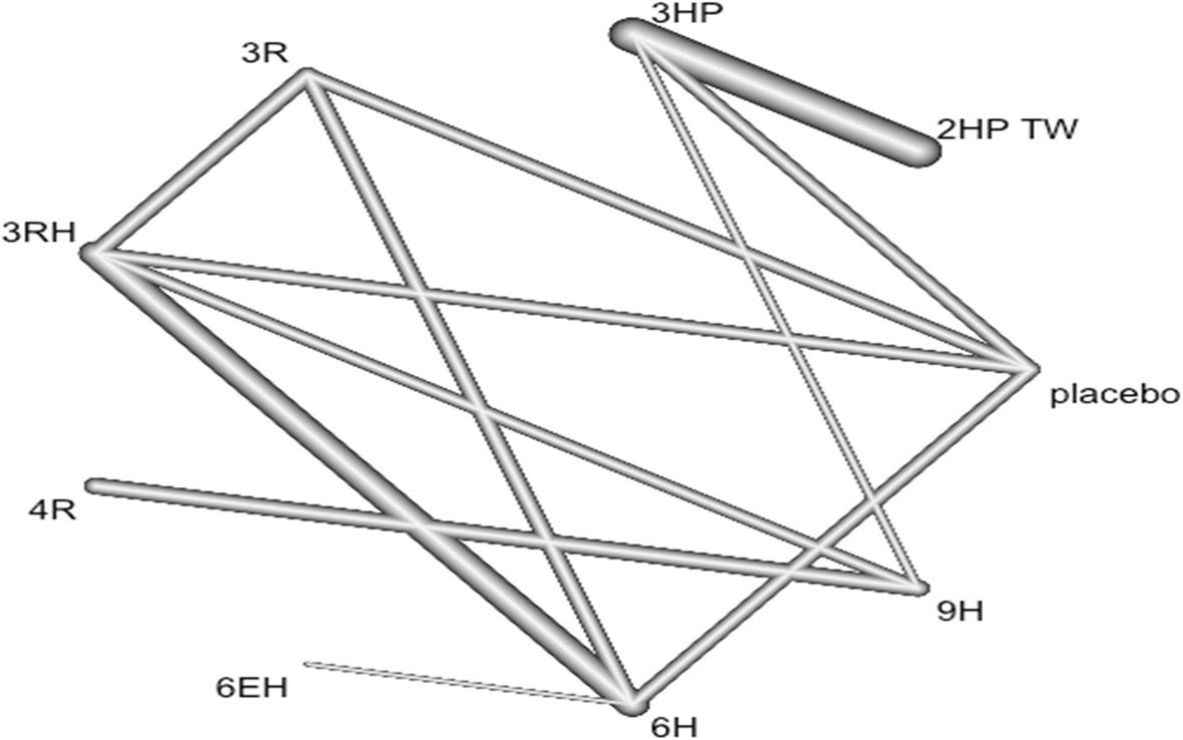
Network diagram for risk of nausea and vomiting among patients who have taken TPT (RCTs published between 1993–2022)

In this analysis 12 studies and 9 treatments were included. The test for a full design-by-treatment interaction random effects mode shows that there is no inconsistency between designs (Q = 7.66 and P-value = 0.053). Nausea and vomiting were more common with patient who have been treated with 3 months weekly combination of isoniazid and rifapentine (3HP) (RR 5.91 95% CI 2.30–15.20), followed by two months twice weekly combination of isoniazid and rifapentine (2HP TW) Fig. 14. However, it was less common among patients who were treated with 4 months daily refampin and nine months daily isoniazid. The network diagram shows that most studies compared 3HP with two months twice weekly HP (2HP TW) Fig. 15.

### Skin rash

**Fig. 16:**
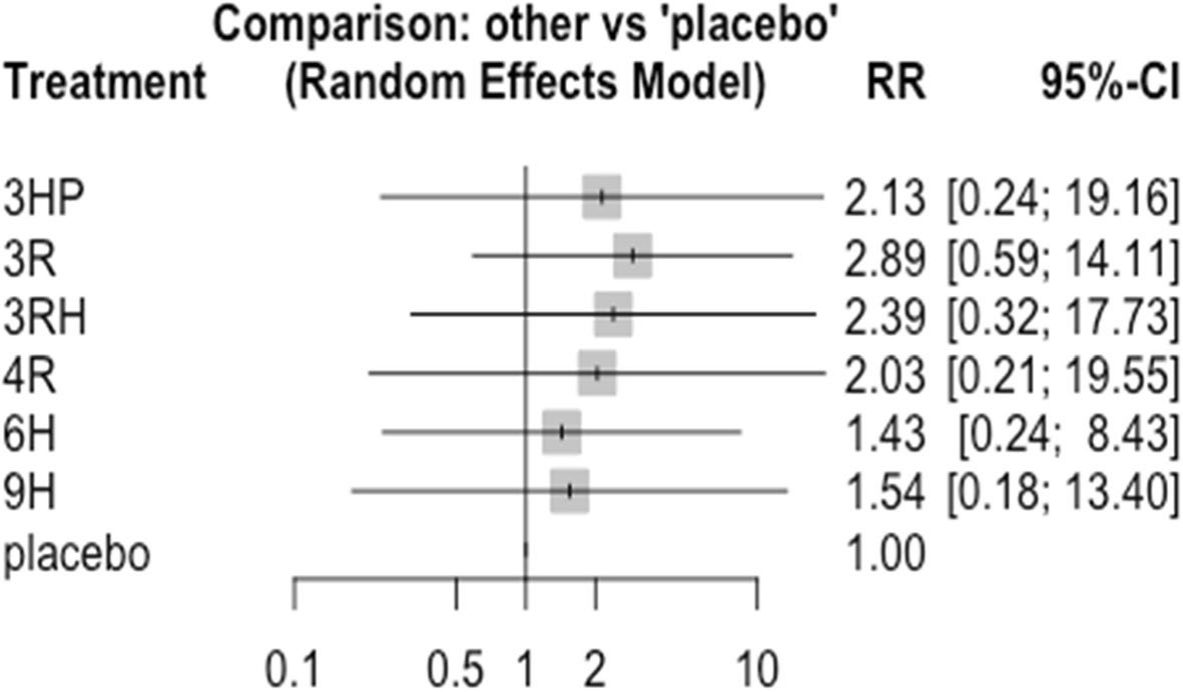
Forest plot for AE (skin rash) among patients who have taken TPT (RCTs published between 1993–2022)

**Fig. 17:**
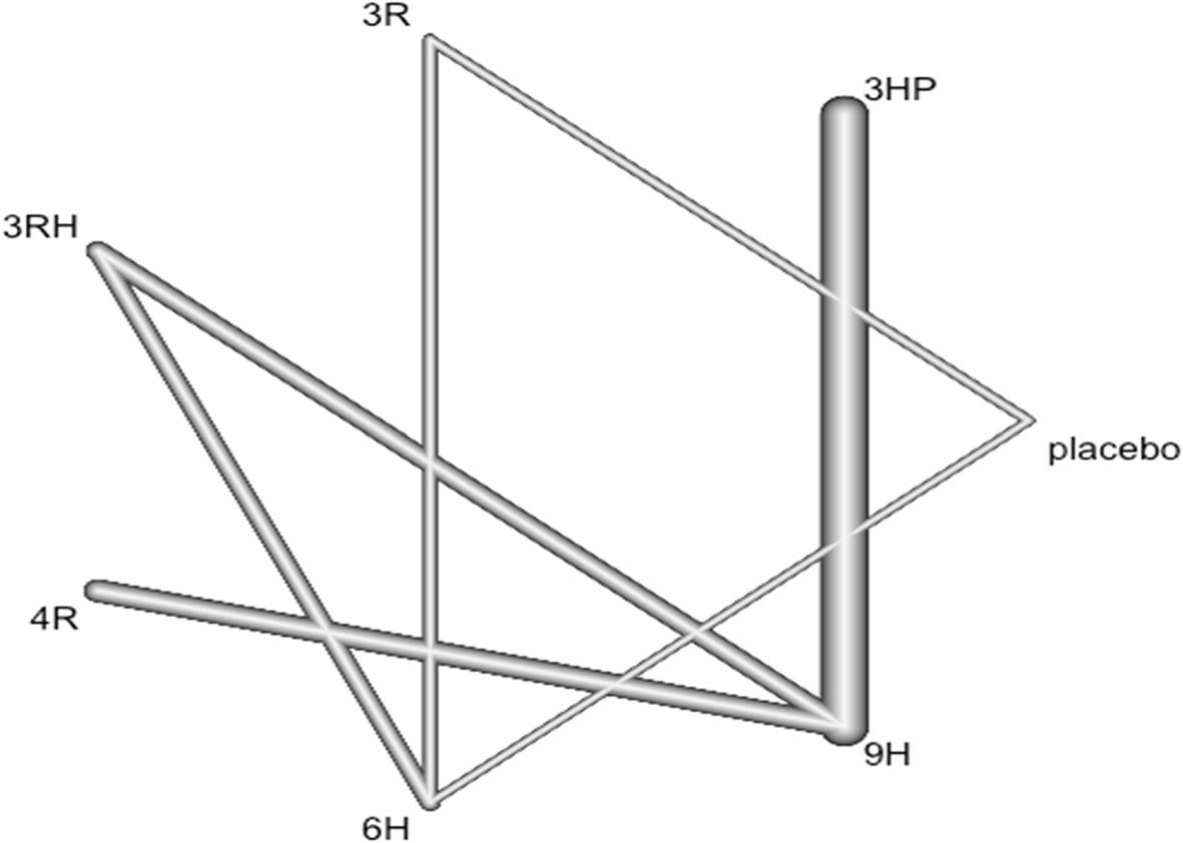
Network diagram for AE (skin rash) among patients who have taken TPT (RCTs published between 1993–2022)

In this analysis 9 studies and 7 treatments were included. The risk of skin rash has no significant difference between the included treatments Figs. 16 and 17.

### Flu like symptom

**Fig. 18:**
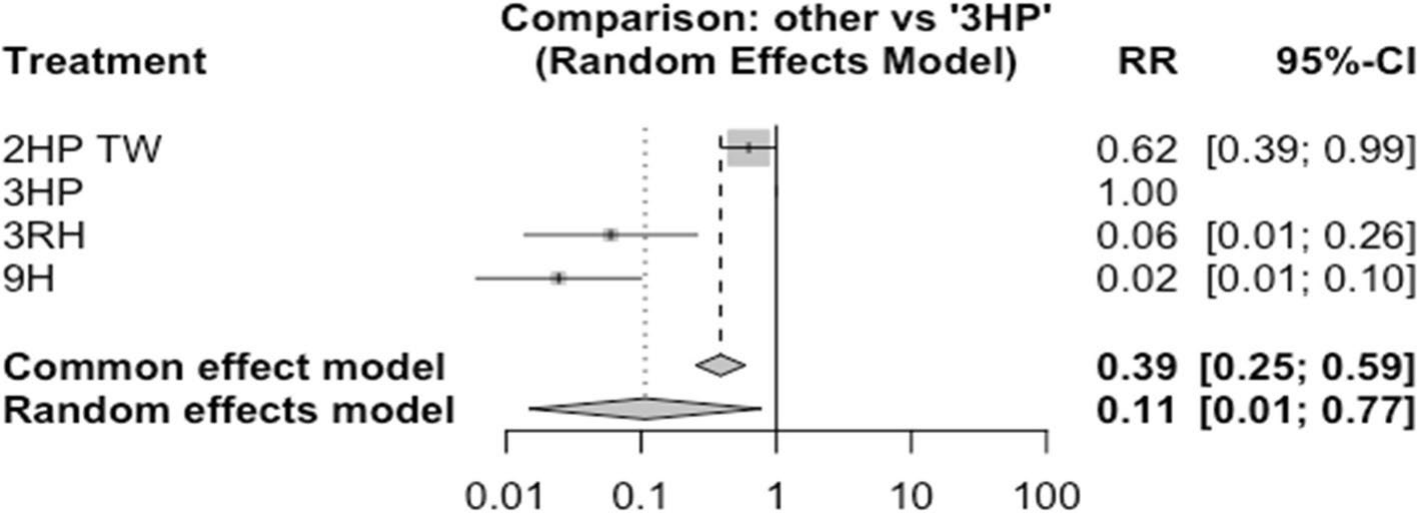
Forest plot for AE (Flu like symptom) among patients who have taken TPT (RCTs published between 1993–2022)

**Fig. 19:**
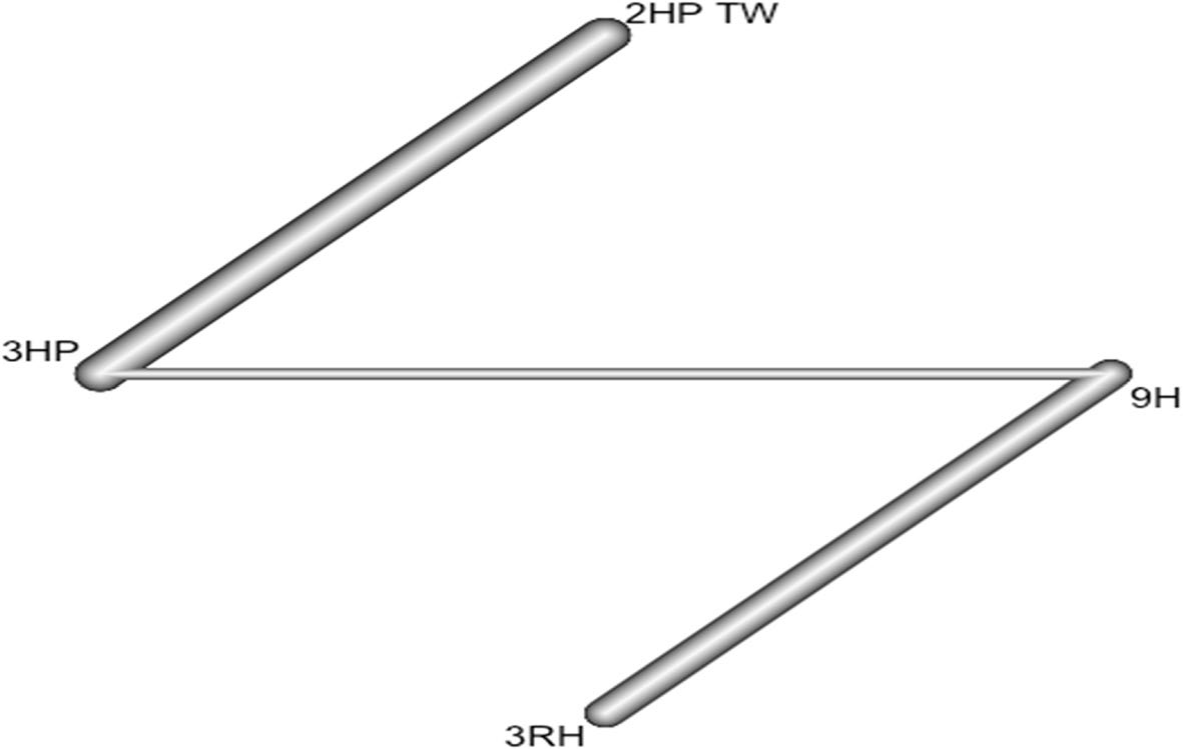
Network diagram for AE (Flu like symptom) among patients who have taken TPT (RCTs published between 1993–2022)

In this analysis 3 studies and 4 treatments were included. There was no inconsistency between designs. Flu like symptom was more common in patients who was were treated with all the included treatments Fig. 18 and majority of the studies compared 3HP with two months twice weekly combination of HP Fig. 19.

### Hypersensitivity reaction

**Fig. 20:**
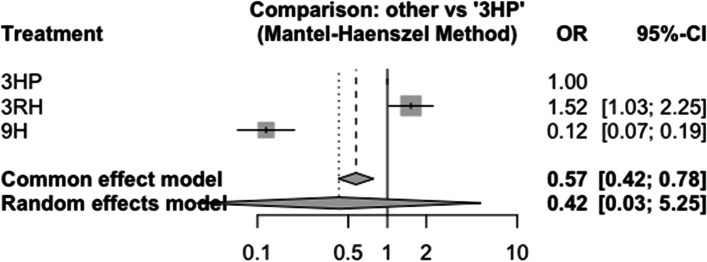
Forest plot for AE (Hypersensitivity reaction) among patients who have taken TPT (RCTs published between 1993–2022)

**Fig. 21:**
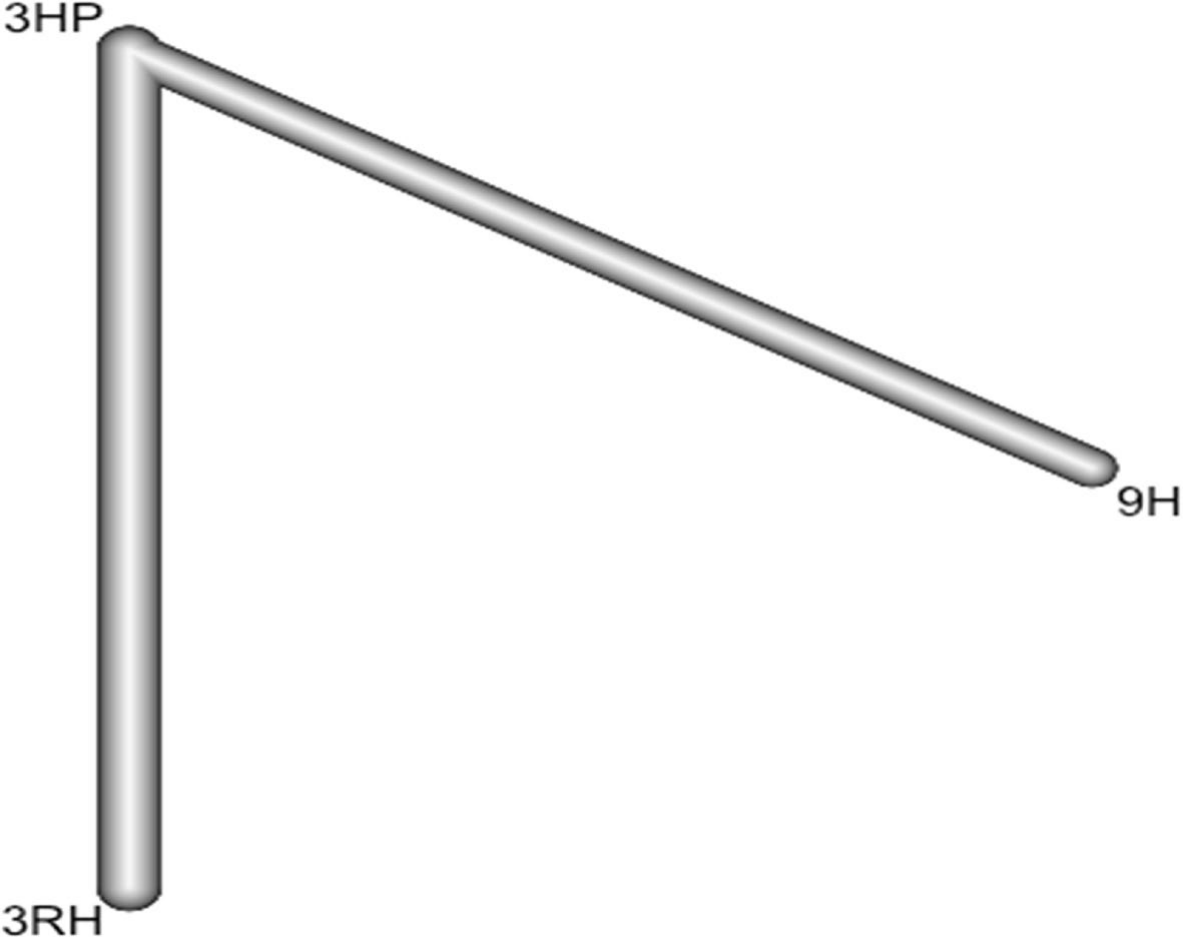
Netwrok diagram for AE (Hypersensitivity reaction) among patients who have taken TPT (RCTs published between 1993–2022)

In this analysis two studies and three treatments were included. There was no inconsistency between designs. Among other adverse events attributed to a study drug, the proportion of subjects with possible hypersensitivity was higher in the 3 months daily combination of rifampin and isoniazid (3RH) Fig. 20 and most of the studies compared 3HP with 3RH Fig. 21.

### Grade III and IV liver toxicity

**Fig. 22:**
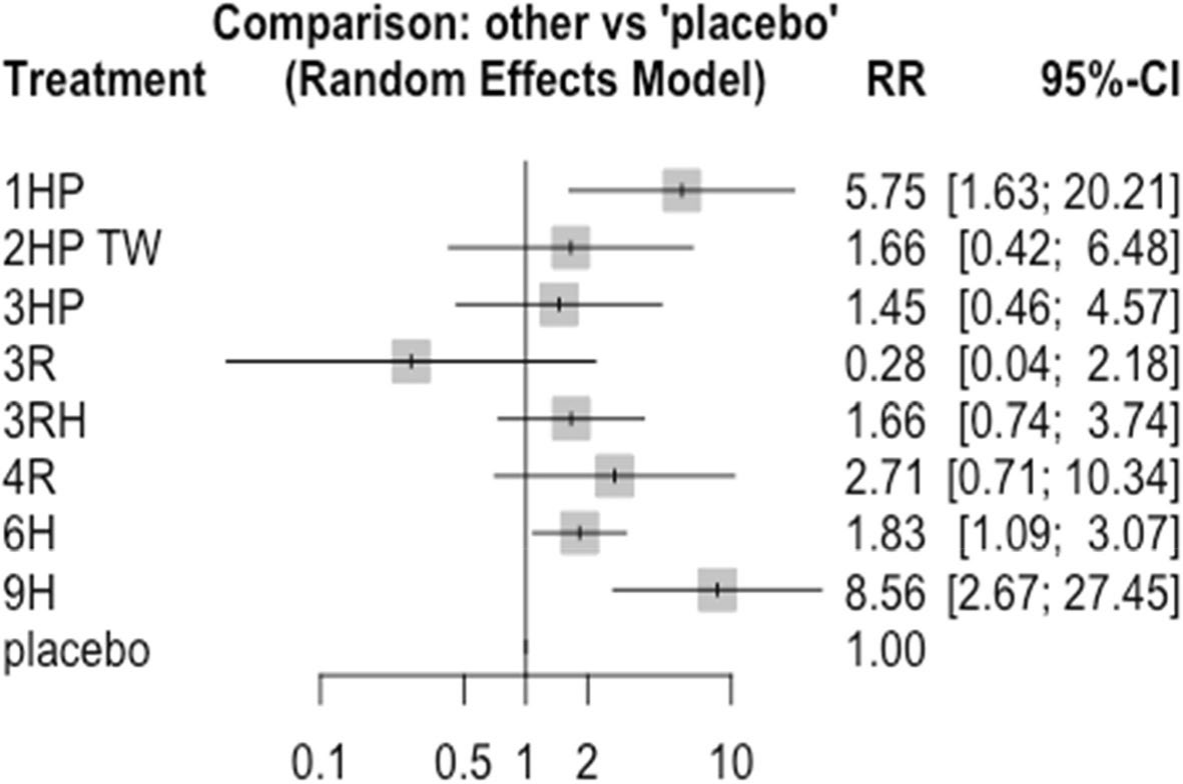
Forest plot for AE (Grade 3 and 4 liver enzyme elevation) among patients who have taken TPT (RCTs published between 1993–2022)

**Fig. 23:**
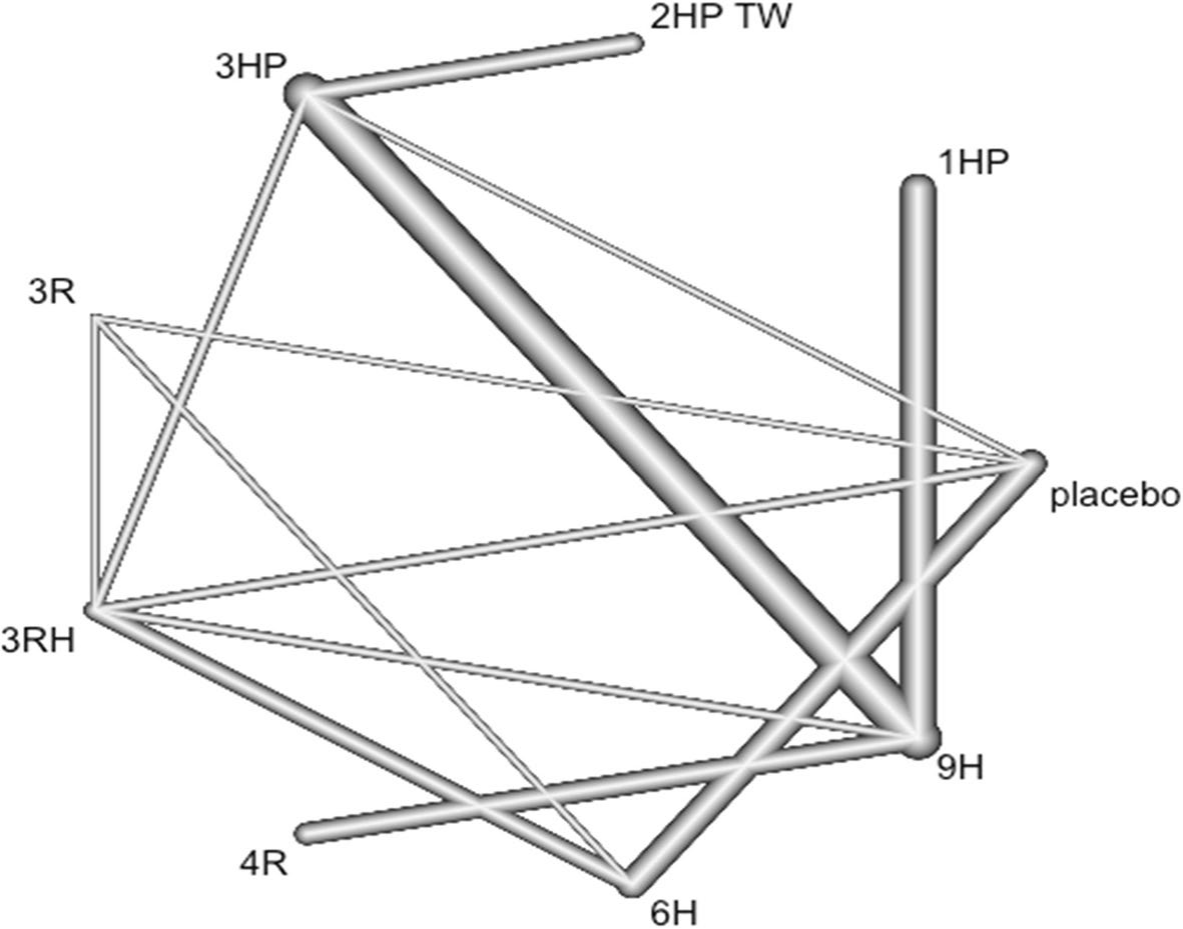
Network diagram for AE (Grade 3 and 4 liver enzyme elevation) among patients who have taken TPT (RCTs published between 1993–2022)

In this analysis 17 studies and 9 treatments were included. The test for inconsistency using a full design-by-treatment interaction random effects model shows that there is no inconsistency between designs (Q = 2.41, tau = 0, tau^2^ = 0, I^2^ = 0%, P = value = 0.66). Liver enzyme elevation after TPT initiation have been noticed in many patients. However, the proportion of subjects with hepatotoxicity that was attributed to a study drug was higher in the 9 months daily isoniazid, followed by 1HP, and 6H. On the contrary, it was less common among patients who have been treated with 3 months daily rifampin Fig. 22. The network diagram shows that majority of the studies were compared 9H with 3HP and 1HP Fig. 23.

### Serious adverse events

**Fig. 24:**
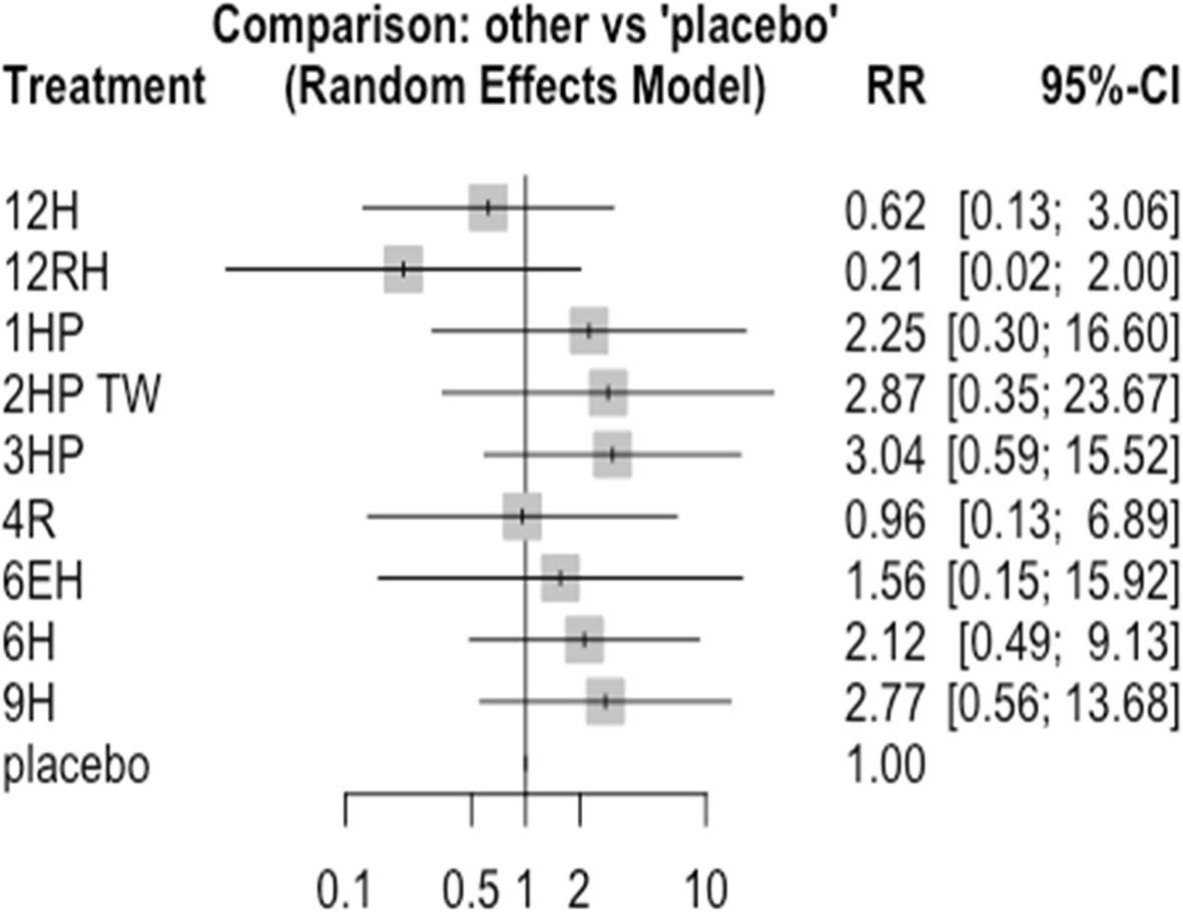
Forest plot of SAEs related to TPT among patients who have taken TPT (RCTs published between 1993–2022)

**Fig. 25:**
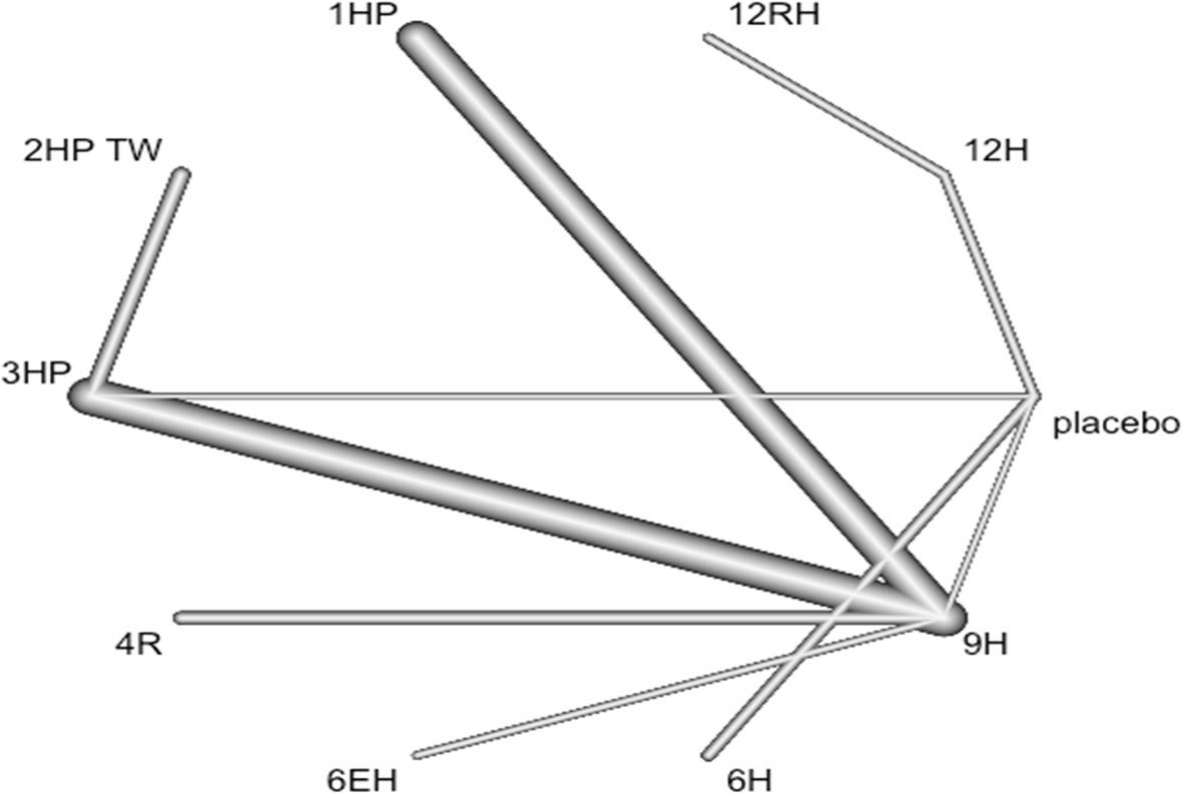
Network diagram of SAEs related to TPT among patients who have taken TPT (RCTs published between 1993–2022)

In this analysis 14 studies and 11 treatments were included. The test for heterogeneity between designs shows moderate inconsistency (I^2^ = 69.9%). However, a test for a full design-by-treatment interaction random effects model shows that there is no inconsistency between designs (Q = 0.3, and P-value = 0.58). Some patients from all treatment groups experienced SAEs, however, the risk of serious adverse events has no statistical difference between the included studies. But, the Net-rank result shows that the frequency of SAES were more common in patients who have been treated with 3HP and 9H. However, it was less common in patients who have been treated with 12 months daily combination of rifampin and isoniazid Fig. 24. The forest plot also shows that there was no significant difference on the risk of SAEs between the treatments and network graph shows that more studies compared 9H with 3HP and 1HP Fig. 25. Furthermore, grade three bilirubin level elevation was higher in 3RH and 12H [15, 25, 31].

## Discussion

Ttreatment of individuals with LTBI is considered a fundamental strategy for the control of TB. Prevention of TB by treating individuals with LTBI is a cost-effective intervention when directed at those with the greatest likelihood of TB, such as recently infected cases, individuals with untreated residual lesions or immuno suppression, children, and recent immigrants from highly endemic regions [59]. Globally, tuberculosis is the principal cause of death in up to one third of people dying who have HIV infection [60-62]. Patients with human immunodeficiency virus (HIV) infection and latent tuberculosis are at substantial risk for the development of active tuberculosis [59]. Many people living with HIV are still dying from TB, despite the availability of ART and measures to control opportunistic infections such as TB are especially important. The prevention of tuberculosis in people with HIV infection has both clinical and public health importance [59].

In this study, the incidence of TB among people living with HIV who have taken 3RH as TPT was lower followed by 6H. However, 3HP shows a significant reduction on the incidence of TB among HIV negative patients who had TB contact history. In terms of preventing TB among PLWHIV, while initiating TPT, it is necessary to consider potential harm such as hepatotoxicity and development of drug resistance, and acceptability of the selected regiment by the patient [3, 30]. A previous network meta-analysis has reported that ﻿all regimens of interest except 9H showed significant benefits in preventing active TB compared to placebo [29]. On the contrary, another previous network meta-analysis reported that 6 to 12 months of isoniazid were no more efficacious in preventing microbiologically confirmed TB than rifamycin-containing regimens [30]. But, a previous meta-analysis also reported that ﻿prolonged regimens (prolonged H and 6H) were more effective in preventing TB [63-65]. However, a 12-dose regimen of once-weekly isoniazid and rifapentine has been shown to be noninferior to 9 months of daily isoniazid in a large and well conducted clinical trial [66]. It has also showed a significant benefits in preventing the development active TB among patients who had a household TB contact history, immigrant population, and old people [16, 19, 67]. Perhaps, those studies which assessed the efficacy of prolonged isoniazid regimens, followed the patients for a short period of time after treatment; thus, the incidence of TB cases detected are only those patients who developed TB while on treatment or shortly after. Yet, difference on the post-treatment follow up time could also have an impact on the incidence of TB among patients. Other factors including the setting where the patients were living (e.g., places where TB incidence were higher), tuberculin skin test positive (ie, ≥ 5 mm induration) at enrolment, high fat intake with TPT regimens, low CD4^+^ cell count, drug addiction, and high ART coverage could also be a confounding factors [3, 30, 65, 68-70].

In order to enhance the potential benefit of TPT and achieve the anticipated efficacy level, patients’ adherence to TPT needs to be higher. The efficacy, adherence, and safety of TPT depends in treatment regimen selection [59]. In this study, patients’ adherence to TPT was higher among patients who have taken 4R followed by 3RH. According to the result from previous network meta-analysis, even if the definitions of regimen completion varied across studies, regimens of 3–4 months were associated with a greater likelihood of adequate completion [29]. ﻿Consistently, a previous study [21] reported that, compared with the 9H regimen, the 3HP regimen had a higher completion rate with lower hepatotoxicity and well-tolerated adverse drug reaction [18]. Most of the previous studies associated higher compliance rate with shorter duration of regimen, drug tolerability, self-limiting adverse drug reactions (ADRs), directly observed administration, and dosing schedule, these factors could affect the completion rate [18, 71]. Other factors such as difference in population group (e.g., prisoners and other marginalized communities), precarious social and economic situation, the diagnosis resulting from screening, which suggests a lack of patient motivation, as there was no known TB focus could also have an impact on the completion rate. In immigrant population, precarious employment, economic difficulties, lack of family support and language and cultural barriers all make patient follow-up more difficult. As this was a healthy population that had immigrated with the intention of working, worrying about their health was not a priority.

The proportion of subjects who permanently discontinued a study drug because of adverse event were higher in patients who has taken 3RH. The frequency of nausea and vomiting were higher among patients who have taken 3HP, followed by two months twice a week combination of HP. However, a previous study reported that, compared with 9H and 3HP had associated with low hepatotoxicity and well-tolerated adverse drug reaction [18, 72]. Furthermore, the risk of grade 3 and 4 liver toxicity was significantly higher among patients who have taken 12H, followed by 2RZ. The risk of hepatotoxicity could be related to prolonged isonizide therapy [18, 30] and combination of isoniazid either with rifampicin [73], age, female sex, white race, non-Hispanic ethnicity, decreased body mass index, alcohol consumption, and elevated baseline AST [16, 74, 75]. Other factor such as frequent liver monitoring and symptom-driven monitoring for hepatotoxicity, could also affect the result [21, 23, 74]. In addition, Heptitis C Virus (HCV) infection was also associated with hepatotoxicity when controlling for other factor [76]. Consistently, a previous study also reported that the risk of hepatitis in patients receiving pyrazinamide/rifampin for prevention of latent tuberculosis is increased threefold as compared to patients receiving isoniazid [75].

### Study limitation

This study has some limitations. Majority of the included studies were conducted in adult patients living with HIV, people who had recent contact with patients with active tuberculosis, evidence radiological of previous tuberculosis, tuberculin test equal or greater than 5 mm, radiographs that indicated inactive fibrotic or calcified parenchymal and/or lymph node lesions, had conversion to positive results on a tuberculin skin test, participants living with HIV, chronic Silicosis, immigrants, prisoners, old people, and pregnant women who were at risk for latent TB. The result of this study might not be a representative of children who either live with HIV or had household TB contact history.

## Conclusion

From this review, it can be concluded 3RH and 6H has a significant impact on the reduction of TB incidence among PLWH and 3HP among HIV negative people who had TB contact history. However, combinations of rifampicin either with isoniazid were significantly associated with adverse events which resulted permanent discontinuation among adult patients. Furthermore, Grade 3 and 4 kiver toxicity was more common in patent’s who have taken 9H, 1HP, and 6H. This may support the current recommended TPT regimen of 3HP, 3RH, and 6H.

## Data Availability

All relevant data are within the manuscript and its supporting information files.

## Abbreviations

AE: Adverse event
CENTRAL: Cochrane Central Register of Controlled Trials
CI: Confidence interval
CINeMA: Confidence in Network Meta-Analysis
E: Ethambutol
EH: Ethambutol Isoniazid combination
GADE: Grading of Recommendations, Assessment, Development, and Evaluations
H: Isoniazid
HP: Isoniazid plus Rifapentine
LTBI: Latent Tuberculosis Infection
NMA: Network meta-analysis
PICO: Population, Intervention, Comparison, and outcome
PLWHIV: Patient Living with Human Immune Virus
PRISMA: Preferred Reporting Items for Systematic Reviews and Meta-Analyses
R: Rifampicin
RCTs: Randomized Control Trials
RH: Rifampicin Isoniazid combination
SAE: Serious Adverse Event
TB: Tuberculosis
TPT: Tuberculosis Prevention Therapy
WHO: World Health Organization
